# An Update to the WISP-1/CCN4 Role in Obesity, Insulin Resistance and Diabetes

**DOI:** 10.3390/medicina57020100

**Published:** 2021-01-23

**Authors:** Małgorzata Mirr, Maciej Owecki

**Affiliations:** Department of Public Health, Poznan University of Medical Sciences, Rokietnicka St. 4, 60-806 Poznań, Poland; malgorzata.tapolska@gmail.com

**Keywords:** CCN4 protein, human, insulin resistance, obesity, diabetes mellitus, type 2, adipokine

## Abstract

Insulin resistance refers to the diminished response of peripheral tissues to insulin and is considered the major risk factor for type 2 diabetes. Although many possible mechanisms have been reported to develop insulin resistance, the exact underlying processes remain unclear. In recent years, the role of adipose tissue as a highly active metabolic and endocrine organ, producing proteins called adipokines and their multidirectional activities has gained interest. The physiological effects of adipokines include energy homeostasis and insulin sensitivity regulation. In addition, an excess of adipose tissue is followed by proinflammatory state which results in dysregulation of secreted cytokines contributing to insulin resistance. Wingless-type (Wnt) inducible signalling pathway protein-1 (WISP-1), also known as CCN4, has recently been described as a novel adipokine, whose circulating levels are elevated in obese and insulin resistant individuals. Growing evidence suggests that WISP-1 may participate in the impaired glucose homeostasis. In this review, we characterize WISP-1 and summarize the latest reports on the role of WISP-1 in obesity, insulin resistance and type 2 diabetes.

## 1. Introduction

Insulin resistance refers to the diminished response of peripheral tissues to insulin and is considered the major risk factor for type 2 diabetes [1]. Although many possible mechanisms have been reported to develop insulin resistance, the exact underlying processes remain unclear [2]. In recent years, the peptides secreted by the adipose tissue and their contribution to glucose homeostasis have gained interest [3]. Since the leptin discovery, the list of adipocytokines has continued to expand. It is currently believed that adipocytes can be the source of more than 600 secretory proteins, most of whose regulatory effects are poorly understood [4]. Wingless-type (Wnt) inducible signalling pathway protein-1 (WISP-1), also known as CCN4, is a matricellular protein that in humans is encoded by the WISP-1 gene located on chromosome 8 [5]. WISP-1 has recently been described as a novel adipokine, which may participate in the impaired glucose homeostasis [6]. In this review, we characterize WISP-1 and summarize the latest reports on the role of WISP-1 in obesity, insulin resistance, and type 2 diabetes.

## 2. The CCN Family

Wnt-induced secreted protein 1 belongs to the secreted extracellular matrix-associated proteins described as the CCN family [6]. Six proteins belong to the CCN family, and each of them has been assigned the acronym CCN with the appropriate number from 1 to 6 [7]. The name of the CCN family comes from the first letters of names of the first three member proteins discovered: Cysteine-rich angiogenic inducer 61 (CYR61), connective tissue growth factor (CTGF) and nephroblastoma overexpressed (NOV). According to this nomenclature, the first three member proteins are also called CCN1, CCN2, and CCN3, respectively [7,8]. The rest of the family, CCN4, CCN5 and CCN6, are represented by the other three proteins: WISP-1, WISP-2 and WISP-3 [7]. The six CCN family members are cysteine-rich proteins and share a structural model characterized by a N-terminal secretory signal, four conserved structural domains with a similar sequence to insulin-like growth factor binding protein (IGFBP), von Willebrand factor type C repeat domain, and thrombospondin type 1 repeat domain, and finally a C-terminal sequence with partial identity to von Willebrand factor [8]. All of them are secreted into the extracellular space, constituting an extracellular ligand participating in various signalling pathways [9,10]. CCN proteins have multidirectional biological functions, including cell growth, differentiation, proliferation, apoptosis, adhesion and migration of multiple cell types [11,12]. CCN proteins participate in the regulation of various physiological processes such as skeletal development, stem cell differentiation, angiogenesis, chondrogenesis or wound repair [12,13]. Abnormal secretion of CCN proteins has been reported in fibrogenesis, carcinogenesis, and atherosclerosis [10,12,14]. CCN proteins interact with various molecules, including proteoglycans, integrins and lipoprotein receptor-related proteins (LRPs), which exerts diverse effects acting both as stimulants or inhibitors in cellular processes [13].

## 3. WISP-1 Multiple Functions

WISP-1 has been associated with oncogenesis, and for this reason, it may have some influence on cancer development and progression. In particular, WISP-1 initially gained interest as a product of the oncogene Wnt1 when it was observed that its expression is increased in colon cancer cell lines and human colon tumours [15]. The role of WISP-1 in tumorigenesis has been studied in many types of neoplasms, where it acts as an autocrine and paracrine modulator, increasing cell migration [10]. Furthermore, the WISP-1 gene’s polymorphisms were shown to impact the breast cancer susceptibility and the expression of oestrogen and progesterone receptors in the tumour [16].

In contrast to its influence on cancer development, WISP-1 may play some protective role in bones. It has been demonstrated that WISP-1 may participate in promoting differentiation of perivascular stem cells into bone cells [9]. Wnt-1 signalling is also considered one of the critical mediators of subchondral bone remodelling, interacting with the OPG-RANK-RANKL pathway [17]. The expression of WISP-1 was noted at sites of new bone formation and fractures healing [18]. For this reason, WISP-1 has been proposed as the possible target for osteoarthritis therapy as it may be involved in stimulating synovial inflammation, cartilage damage, and osteophytes forming [18].

Apart from cancer and bone, WISP-1 may influence the processes of fibrosis and apoptosis, which has been demonstrated in particular in the kidney, lungs, heart and liver. In the kidney, WISP-1 is also associated with renal fibrosis as the elevated serum level of WISP-1 has been noted in biopsy-proven renal fibrosis as compared to normal patients [19]. The mechanism of this finding was investigated by Yang et al. and it was suggested that WISP-1 stimulated the production of transforming growth factor beta 1 (TGF-beta-1) induced profibrotic markers [20]. Furthermore, WISP-1 was upregulated in idiopathic pulmonary fibrosis and in bleomycin-induced lung fibrosis in mice [21]. Interestingly, the orotracheal application of WISP-1 neutralizing antibodies to the lung has been shown to decrease bleomycin-induced lung fibrosis [21], suggesting that WISP-1 might be a new target for anti-fibrotic therapy [22]. Similarly, the possible contribution of WISP-1 in cardiac fibroblast proliferation in postinfarct myocardium was proposed [23]. In addition, WISP-1 was identified as an autocrine angiogenic mediator for human coronary artery endothelial cells [24]. Venkatachalam et al. suggested that WISP-1 stimulates fibroblast proliferation in the myocardium but also inhibits tumor necrosis factor alpha-induced cardiomyocyte death, thereby suggesting some protective role of WISP-1 in the heart [25]. The amount of WISP-1 was significantly higher in human coronary arteries with early intimal thickening compared with normal control arteries. WISP-1 was shown to promote vascular smooth muscle cells migration and thereby intimal thickening [26]. The profibrotic activity of WISP-1 raised interest in the possible role of this mediator in the liver fibrosis [27]. The upregulation of WISP-1 in both in vivo and in vitro liver fibrosis models has been demonstrated [27].

## 4. WISP-1 and Obesity

In recent years, the role of WISP-1 as an adipokine has gained interest [6] and Wnt signaling has been shown to participate in human adipogenesis [28]. Based on mouse studies, Wnt signalling may regulate adipose tissue distribution [28]. In line with these results, Murahovschi et al. conducted a study to validate WISP-1 as an adipokine and demonstrated WISP-1 was secreted by fully differentiated human adipocytes [6]. In addition, a reduction of WISP-1 mRNA expression in subcutaneous adipose tissue in female mice after a weight loss was observed [6]. WISP-1 expression was increased after weight gain in male high fat diet-fed mice compared with controls [6].

Studies show that circulating WISP-1 levels were significantly higher in obese men than in normal-weight men independently of diabetes status [29]. Another study showed that serum WISP-1 level positively correlated with body mass index, body fat percentage, leptin and triglyceride levels, hip circumference and fatty liver index [11]. However, in the same study, the differences in the WISP-1 levels between normal weight, overweight and obese subjects did not reach statistical significance [11]. In the study conducted by Barchetta et al., WISP-1 serum levels increased throughout the obesity class and correlated with visceral adipose tissue area assessed with magnetic resonance imaging [30]. The relationship between WISP-1 and obesity was also investigated in a group of children. Serum levels of WISP-1 were found higher in the group of obese children and adolescents compared with the normal weight healthy controls [31]. WISP-1 level correlated positively with body mass index (BMI) and BMI z-score in the group of obese children [31].

## 5. WISP-1 in Adipose Tissue and Systemic Inflammation

Since the excess of adipose tissue is followed by a proinflammatory state, which promotes insulin resistance and obesity-derived disorders, the relationship between WISP-1 and inflammation was studied [6,32]. It has been shown that WISP1 mRNA expression in subcutaneous and visceral adipose tissue correlates with macrophage infiltration [6]. A dose-dependent WISP-1 induced increase of mRNA of proinflammatory cytokines was observed. Furthermore, WISP-1 altered macrophages’ polarization toward the M1 proinflammatory phenotype [6]. Barchetta et al. demonstrated that WISP-1 plasma concentration correlates with interleukin 8 (IL-8) levels. The patients with increased plasma IL-6 and tumor necrosis factor alpha (TNF-alpha) levels presented significantly higher WISP-1 levels [30].

Furthermore, Jung et al. investigated the mechanisms by which WISP-1 may contribute to inflammation in the pathogenesis of non-alcoholic fatty liver disease and insulin resistance [33]. The study showed that WISP-1 knockdown in high-fat diet-fed mice reduced proinflammatory markers. Moreover, the treatment of mouse hepatocytes with recombinant WISP-1 caused the concentration-independent increase in inflammatory markers’ levels [33]. Finally, the study by Wang et al. showed that WISP-1 concentration correlated with IL-18, which is a strong proinflammatory cytokine [31].

## 6. WISP-1 and Insulin Resistance

Impaired adipose tissue function is considered a fundamental pathogenetic mechanism of insulin resistance [34]. It has been demonstrated that altered adipokines release, oxidative stress, chronic inflammation state in non-adipose tissues are major factors contributing to impaired insulin sensitivity [34]. Since WISP-1 circulating concentrations and expression in adipocytes are associated with obesity and inflammatory markers, a contribution of WISP-1 in insulin resistance development has become the subject of research. 

WISP-1 expression in subcutaneous and visceral adipose tissue correlates positively with fasting insulin and negatively with insulin sensitivity, assessed as glucose infusion rate in the euglycemic-hyperinsulinemic metabolic clamp [6]. A negative correlation between WISP-1 mRNA expression and circulating adiponectin was observed [6]. It was demonstrated that the gene expression of WISP-1 was increased after insulin stimulation in vitro. In the same study, WISP-1 expression in adipose tissue was not altered after insulin stimulation in vivo [6]. However, this observation was performed in a group of overweight glucose-tolerant individuals [6]. A study by Hörbelt et al. showed that circulating WISP-1 levels were positively associated with blood glucose levels in oral glucose tolerance test and confirmed a negative correlation between serum concentrations WISP-1 and adiponectin [29]. In primary human skeletal muscle cells and murine AML12 (alpha mouse liver 12) hepatocytes, recombinant WISP-1 impaired insulin action by inhibiting phosphorylation of insulin receptor, Akt and glycogen synthase kinase 3β, and inhibiting insulin-stimulated glycogen synthesis and suppression of gluconeogenic genes [29].

The influence of exercise on WISP-1 serum concentrations was studied in the group of breast cancer survivors [35]. The reduced WISP-1 levels, together with the improvement of the gluco-lipid profile after a 12-week exercise were noted compared to the control group [35]. A significant correlation between changes in Homeostatic Model Assessment of Insulin Resistance (HOMA-IR) and WISP-1 concentrations was observed [35]. Multivariate analysis demonstrated that body mass index, HOMA-IR, and anti-Müllerian hormone independently and positively predicted WISP-1 levels in the group of women diagnosed with polycystic ovary syndrome [36]. Circulating WISP-1 levels were found higher in the group of women with gestational diabetes mellitus compared to healthy controls. WISP-1 correlated positively with BMI, HOMA-IR values, fasting glucose, fasting insulin, and triglyceride concentrations [37].

Moreover, in the study conducted by Jung et al. treatment with WISP-1 increased lipogenesis-associated gene expression and accumulation of triglycerides in murine hepatocytes and inhibited insulin signaling in murine skeletal muscle cells [33]. The results indicate that WISP-1 promotes insulin resistance through the Toll-like receptor 4 (TLR-4)-mediated and c-Jun N-terminal kinase (JNK)-dependent pathway [33].

## 7. WISP-1 and Diabetes

As WISP-1 seems to play a role in obesity and insulin resistance, it may hypothetically also affect glucose homeostasis, thereby having some plausible impact on the development of diabetes mellitus. However, to date, conflicting reports exist, and no clear evidence to support this hypothesis has been demonstrated. Several studies investigated the association of WISP-1 and type 2 diabetes mellitus. Klimontov et al. demonstrated significantly higher serum levels of WISP-1 in the group of subjects diagnosed with type 2 diabetes mellitus than in healthy controls [38]. In contrast, a study comparing circulating levels of WISP-1 in the group of men with obesity with and without type 2 diabetes mellitus revealed no significant difference [29]. Similarly, a study by Tacke et al. presented no difference in WISP-1 concentrations between individuals with normal glucose tolerance and the group with type 2 diabetes mellitus [11]. However, the authors suggested the results may be explained by the good therapeutic glycaemic control of the group with diabetes [11]. In agreement with these findings, WISP-1 concentration did not differ significantly between participants with and without type 2 diabetes in the research by Barchetta et al. [30]. It appears that while the higher plasma concentration of WISP-1 is associated with insulin resistance, it is not further increased with type 2 diabetes [29]. Thus far, there have been no reports directly linking WISP-1 with type 1 diabetes mellitus.

## 8. WISP-1 and Beta Cell Biology

Since the loss of beta cells is the major pathogenetic factor of diabetes, Fernandez-Ruiz et al. tested the potential of WISP-1 as a beta cell trophic factor [39]. The authors presented that human adult beta cells show increased proliferation when transplanted into pre-weaning mice and identified WISP-1 as enriched in pre-weaning mice relative to adult serum [39]. Wisp1 gene expression in islet cells was negligible, while the bone seems to be the likely source of serum WISP-1 in young mice [39]. The study showed that WISP-1 contributes to beta cell proliferation in young and adult mice [39]. The administration of adenoviral particles encoding the human isoform of WISP1 to diabetic adult mice did not normalize hyperglycaemia, but a significant increase in insulin plasma levels as compared to controls was observed [39].

## 9. Conclusions

WISP-1 can be considered one of the essential adipokines involved in glucose homeostasis. Growing evidence supports the assumption that WISP-1 plays a role in the complex continuum, which consists of excessive body weight, impaired insulin sensitivity and finally, type 2 diabetes. WISP-1 is secreted by differentiated human adipocytes and its circulatory levels are higher in obese patients compared to those with normal weight. The ability of WISP-1 to induce a pro-inflammatory state has been shown, which may be one of the mechanisms enhancing the endocrine dysfunction of adipose tissue. Furthermore, WISP-1 expression in adipocytes negatively correlates with insulin sensitivity.

It appears that the mechanisms of WISP-1 action impairing insulin sensitivity comprise inhibition of glycogen synthesis, interfering with insulin signalling, and promotion of the inflammatory state. Conflicting reports concern the relationship between WISP-1 circulatory levels and type 2 diabetes, which needs clarification. WISP-1 promotes human beta cell proliferation, making it a candidate for future therapeutic use. However, the mitogenic, profibrotic and angiogenic potential of WISP-1 in various tissues poses a risk of undesirable effects. Further rodent or in vitro studies would be valuable to establish the possible effect of WISP-1 on glycaemic control.

Concluding, further studies are needed to precisely determine the role of this promising adipokine in the pathogenesis of insulin resistance and perhaps to discover its possible future use in a therapeutic approach to insulin resistance and obesity.

## Data Availability

Not applicable.
